# *Toxoplasma gondii* infection and testosterone alteration: A systematic review and meta-analyses

**DOI:** 10.1371/journal.pone.0297362

**Published:** 2024-04-03

**Authors:** Amir Abdoli, Fatemeh Ghaffarifar, Zohreh Sharifi, Ali Taghipour

**Affiliations:** 1 Zoonoses Research Center, Jahrom University of Medical Sciences, Jahrom, Iran; 2 Department of Parasitology and Mycology, Jahrom University of Medical Sciences, Jahrom, Iran; 3 Department of Parasitology, Faculty of Medical Sciences, Tarbiat Modares University, Tehran, Iran; 4 Blood Transfusion Research Center, High Institute for Research and Education in Transfusion Medicine, Tehran, Iran; National Institute of Child Health and Human Development (NICHD), NIH, UNITED STATES

## Abstract

**Background:**

*Toxoplasma gondii* (*T*. *gondii*) is a worldwide distributed protozoan parasite which has infected a wide range of warm-blooded animals and humans. The most common form of *T*. *gondii* infection is asymptomatic (latent); nevertheless, latent toxoplasmosis can induce various alterations of sex hormones, especially testosterone, in infected humans and animals. On the other hand, testosterone is involved in behavioral traits and reproductive functions in both sexes. Hence, the purpose of this systematic review is to summarize the available evidence regarding the association between *T*. *gondii* infection and testosterone alteration.

**Methods:**

In the setting of a systematic review, an electronic search (any date to 10 January 2023) without language restrictions was performed using Science Direct, Web of Science, PubMed, Scopus, and Google Scholar. The PRISMA guidelines were followed. Following the initial search, a total of 12,306 titles and abstracts were screened initially; 12,281 were excluded due to the lack of eligibility criteria or duplication. Finally, 24 articles met the included criteria. A mean±standard deviation (SD) was calculated to assess the difference of testosterone between *T*. *gondii* positive and *T*. *gondii* negative humans. The possibility of publication bias was assessed using Egger’s regression. *P*-value < 0.05 was considered statistically significant.

**Results:**

This systematic review identified 24 articles (18 studies in humans and six studies in animals). Most human studies (13 out of 19) reported an increased level of testosterone following latent toxoplasmosis in males, while three studies reported decreased levels and two studies reported an insignificant change. Eleven articles (seven datasets in males and seven datasets in females) were eligible to be included in the data synthesis. Based on the random-effects model, the pooled mean± SD of testosterone in *T*. *gondii* positive than *T*. *gondii* negative was increased by 0.73 and 0.55 units in males and females, respectively. The Egger’s regression did not detect a statistically significant publication bias in males and females (*p* = value = 0.95 and 0.71), respectively. Three studies in male animals (rats, mice, and spotted hyenas) and two studies in female animals (mice and spotted hyenas) reported a decline in testosterone in infected compared with non-infected animals. While, one study in female rats reported no significant changes of testosterone in infected than non-infected animals. Moreover, two studies in male rats reported an increased level of testosterone in infected than non-infected animals.

**Conclusions:**

This study provides new insights about the association between *T*. *gondii* infection and testosterone alteration and identifies relevant data gaps that can inform and encourage further studies. The consequence of increased testosterone levels following *T*. *gondii* infection could partly be associated with increased sexual behavior and sexual transmission of the parasite. On the other hand, declining testosterone levels following *T*. *gondii* infection may be associated with male reproductive impairments, which were observed in *T*. *gondii*-infected humans and animals. Furthermore, these findings suggest the great need for more epidemiological and experimental investigations in depth to understand the relationship between *T*. *gondii* infection and testosterone alteration alongside with future consequences of testosterone alteration.

## 1. Introduction

*Toxoplasma gondii* (*T*. *gondii*) is a worldwide prevalent intracellular protozoan parasite which infects about one-third of human and animal populations [1, 2]. The cat family (Felidae) as the definitive hosts and a wide spectrum of warm-blooded vertebrates including humans serve as intermediate hosts [1, 2]. Humans get the infection through ingestion of contaminated foods and water containing oocytes which shed in the cat feces, or by consumption of raw/undercooked meat containing parasite tissue cysts [2]. Other routes of transmission include organ transplantation and blood transfusion from infected to uninfected individuals, as well as congenital transmission from infected mothers to their fetus [1, 3]. Recent studies also suggested that the parasite could transmit via sexual intercourse in humans [4] and rats [5].

According to estimations, more than one-third of the human population has been infected with the parasite worldwide [2]. Nevertheless, most human infections are asymptomatic in immunocompetent individuals [6]. In immunocompromised individuals, the infection could have life-threatening sequels, such as toxoplasmic encephalitis, myocarditis, or disseminated infections [7, 8]. Congenital toxoplasmosis is also a life-threatening condition which may lead to abortion, stillbirth, and preterm birth [9–12]. The intensity of *T*. *gondii* infection depends on several factors, including genetic background [13], immunity status [14], and the parasite virulence [15, 16]. *T*. *gondii* consists of three main strains (Types I, II, and III), which have some differences in virulence factors and epidemiological patterns [17–19]. While type I strains (such as RH and GT-1) are highly virulent, type II strains (e.g., ME49 and PRU) and type III (e.g., VEG, NED, and CEP) have lower virulence than type I strains [19, 20].

Testosterone is the primary male hormone that is responsible for male sex characteristics and reproductive functions, such as spermatogenesis and fertility. Females also need certain levels of testosterone. In females, most testosterone converts into the sex hormone estradiol [21]. Testosterone is primarily produced in the testes and ovaries in males and females, respectively. A small amount of testosterone is produced in the adrenal glands in both sexes [21].

Several studies in humans and animal models revealed that *T*. *gondii* infection influenced testosterone levels. While some studies reported an increased level of testosterone, others reported a decline level following *T*. *gondii* infection [22]. It seems that several factors, such as the parasite strain and intensity of infection could influence this variation [22]. Inasmuch as testosterone is important in different physiological processes (e.g., reproductive function and sexual behavior), this systematic review is aimed to summarize data regarding the effects of *T*. *gondii* infection on testosterone levels in humans and animals and discusses their influential factors.

## 2. Materials and methods

The present study was conducted following the guideline of the Preferred Reporting Items for Systematic reviews and Meta-Analyses (PRISMA) statement [23] (S1 Checklist).

### 2.1 Strategy search

The search was performed in international databases (Science Direct, Web of Science, PubMed, and Scopus) and the search engine, Google Scholar, published from any date to 10 January 2023. The following search terms were selected using Medical Subject Heading (MeSH) terms alone or in combination: (“*Toxoplasma gondii*” OR “*T*. *gondii*” OR “toxoplasmosis”) AND (“testosterone” OR “hormone” OR “androgen”). Additionally, to avoid ignoring the reference lists of all included studies were reviewed. As such, the citations of all selected articles were hand-searched in Google Scholar for potentially eligible articles.

### 2.2 Eligibility criteria, study selection, and data extraction

Two independent reviewers (AA and AT) selected the articles. After the initial search, all selected articles were screened by title and abstract, then the relevant articles were imported into the EndNote X8 software (Thomson Reuters, New York, USA). Duplicated articles were checked and removed in the next step. Then, if the articles met the following criteria, they were included in the systematic review: (1) papers with full-text or abstract in English, and (2) original research articles, short reports or letters to the editors that studied the association between *T*. *gondii* infection and testosterone. Articles were included if they fulfilled the following Population, Intervention, Comparison and Outcomes (PICO) criteria [24]: Participants/Population: animals or humans, Interventions/exposure: *T*. *gondii* infection, Comparison or control: compared with uninfected human or animals, Outcomes: levels of testosterone.

The data extracted and tabulated from each study, including: (1) First Author, (2) Publication Year, (3) Country, (4) Study Design, (5) Type of Population (case and control), and (6) Findings. All extracted data was entered into the respective tables (for humans and animals) by the primary researcher and verified by another researcher. Any discrepancies were reviewed and resolved by consensus.

### 2.3 Quality assessment

Quality assessment of the included articles was done by The Joanna Briggs Institute (JBI) Critical Appraisal Checklist [25], which contains eight questions with four options including Yes, No, Unclear, and Not applicable. For including and excluding papers, each paper takes a maximum of one star for each numbered item and the total score of 4–6 and 7–10 points were specified as moderate and high quality, respectively. Based on the obtained score, the authors have decided to include (4–10 points) and exclude (≤3 points) the papers.

### 2.4. Data synthesis and statistical analysis

Data was analyzed using comprehensive meta-analysis software version 2. To assess the association between *T*. *gondii* with testosterone in humans, a mean±standard deviation (SD) using the random effects model and corresponding 95% confidence intervals (CI) were calculated for each study. Egger’s regression (Qualitative method) was applied to assess the possibility of publication bias during the analysis. *P*-value < 0.05 was considered statistically significant.

## 3. Results

### 3.1 Study selection

**Fig 1:**
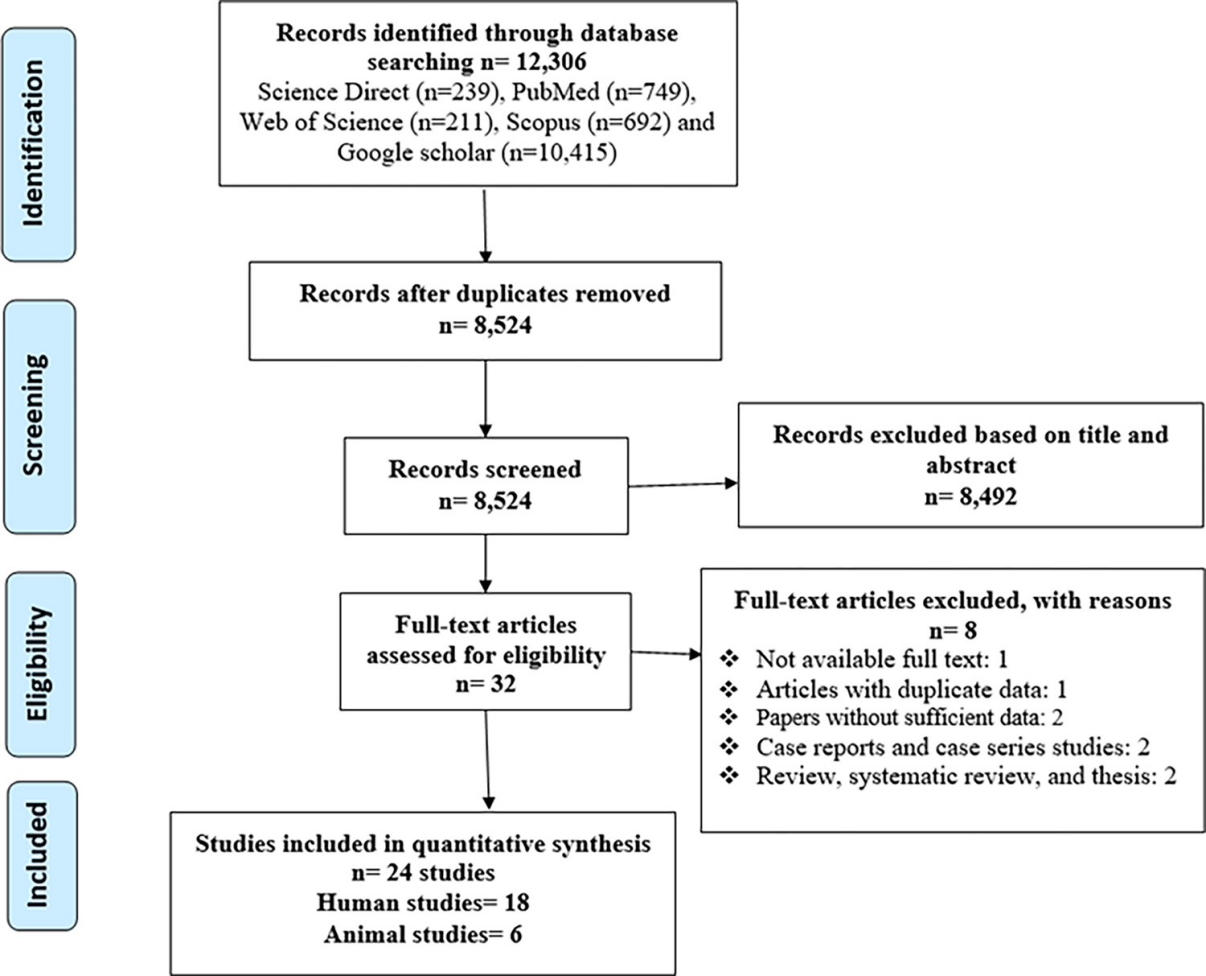
PRISMA flow diagram describing included/excluded studies.

As shown in the PRISMA flowchart (Fig 1), a total of 12,306 titles and abstracts were screened initially; 12,281 were excluded due to the lack of eligibility criteria or duplication. Finally, 24 articles (18 studies in human and six animal studies) met the included criteria. Tables 1 and 2 summarize the information of the included articles regarding the association between *T*. *gondii* infection and testosterone in humans and animals, respectively.

**Table 1:** Association of *T*. *gondii* infection with testosterone alteration in human in the case-control studies.

NO	First Author, Publication Year, and Country	Study aims and design	Studied groups	Findings	QA
1	Flegr et al. [40] 2008 Czech Republic	●A case-control study to assess the relationship between *T*. *gondii* seropositivity with testosterone levels.	●A group of 174 female and 91 male students. ●29 (16.7%) females and 23 (25.3%) males were *T*. *gondii* seropositive.	►*Toxoplasma*-infected **men** have a **higher** concentration of **testosterone** and *Toxoplasma*-infected **women** have a **lower** concentration of **testosterone** than *Toxoplasma*-free controls.	9
2	Flegr et al. [41] 2008 Czech Republic	●A case-control study to test the relationship between *T*. *gondii* seropositivity with testosterone levels and 2D:4D ratio.	●A group of 194 females and 106 males. ●31 (15.9%) females and 25 (23.6%) males were *T*. *gondii* seropositive.	►Infected **males** had **higher** and infected **females** had **lower** testosterone levels than *Toxoplasma*-free males and females, respectively (*P* = 0.007). ►*Toxoplasma*-infected males had a lower left hand 2D:4D ratio than Toxoplasma-free males (*P* = 0.008).	9
3	Shirbazou et al. [26] 2011 Iran	●A case-control study to investigate the relationship between *T*. *gondii* seropositivity with serum cortisol and testosterone levels, as well as with depression, anxiety, and stress index.	●180 people (73 females and 107 males) healthy individuals. ●24 females (13/33%) and 39 males (21/66%) had anti-*Toxoplasma* IgG antibody. ●12 females and 19 males without *Toxoplasma* gondii IgG antibody	►Serum **cortisol** and **testosterone** concentrations were significantly **increased** in seropositive **women and men** compared with seronegative counterparts. ►**Stress and anxiety** index were also **increased** in seropositive men and women, whereas **depression** index **increased** only in seropositive men.	9
4	Abdul-Lateef et al. [32] 2012 Iraq	● A case-control study to investigate the relationship between *T*. *gondii* seropositivity with testosterone, IFN- and IL-12 serum levels.	●77 healthy individuals with anti-*Toxoplasma* antibodies (40 females and 37 males) and 30 (15 females and 15 males) seronegative control	►*T*. *gondii* seropositive **women and men** had significantly **higher** levels of **IL-12, IFN-γ, and testosterone** than seronegative control group.	8
5	Eslamirad et al. [28] 2013 Iran	●A case-control study to investigate the relationship between chronic *T*. *gondii* infection with serum testosterone in men	●1026 healthy men who referred to Arak Post Marriage Center. ●365 men with *T*. *gondii* IgG antibody were selected as case group and 365 seronegative men was selected as control group	►**Testosterone** concentration in case group (seropositive **men**) was **decreased** than control group and this difference was statistically significant (*P*<0.05).	10
6	Eslamirad et al. [27] 2014 Iran	●A case-control study to examine the relationship between *T*. *gondii* infection with serum testosterone and lipid profile in healthy men	●100 men with *T*. *gondii* IgG antibody and equal number of men without *T*. *gondii* antibodies	►**Testosterone** levels level was significantly **lower** among seropositive men ►No significant difference was found between *T*. *gondii* seropositivity and **serum lipid levels**.	9
7	Mahbodfar et al. [29] 2015 Iran	●A case-control study to examine the relationship between *T*. *gondii* infection with serum testosterone, DHEA, cortisol and prolactin among young persons ●The prevalence of hirsutism, acne and alopecia were also investigated	●215 (106 women and 107 men) blood samples (age 18–35 years). ●61 men and 58 women were seropositive for (IgG) *T*. *gondii* antibodies ●47 men and 49 women were seronegative for (IgG) *T*. *gondii* antibodies	►A significant **increase** in **testosterone** and **cortisol** was found in *T*. *gondii* seropositive individuals, but not for DHEA. ►A significantly **increased** in the rates of **alopecia** and **acne** in seropositive **men** than seronegative men. ► A significantly **increased** in the rates of **hirsutism** in seropositive **women** than seronegative women.	10
8	Colosi et al. [42] 2015 Romania	●A case-control study to examine the relationship between *T*. *gondii* infection with male fertility in human	●60 immunocompetent males. ●15 men with *T*. *gondii* IgG antibody ●45 men without *T*. *gondii* IgG antibody	►Serum **testosterone** concentration in seropositive **men** was lower than seronegative individuals, but it was not statistically significant (*P* = 0.62). ►Serum **FSH** concentration in seropositive men was increased than seronegative individuals, but it was not statistically significant (*P* = 0.97). ►**Sperm characteristics** (ejaculate quantity, sperm count, motility, morphology) was not statistically significant changed (*P*>0.05) in *T*. *gondii* infected than noninfected individuals.	10
9	Zghair et al. [33] 2015 Iraq	●A case-control study to investigate the relationship between chronic *T*. *gondii* infection with total testosterone, free testosterone and FSH levels healthy men	●400 apparently healthy blood donor males were evaluated for *T*. *gondii* antibodies. ●10 *T*. *gondii-*IgM positive and 121 *T*. *gondii-*IgG positive were enrolled as case group. ●30 seronegative individuals were enrolled as control group	►Concentrations of total and free **testosterone** were significantly **higher** in both IgM and IgG seropositive men compared with seronegative control group ►Concentration of **FSH** was not significant differences in both IgM and IgG seropositive men compared with seronegative control group	10
10	Zouei et al. [30] 2018 Iran	●A case-control study to investigate the relationship between *T*. *gondii* seropositivity with serum testosterone in men and women	●76 positive sera were selected as case group (38 from men and 38 from women) and a same number of negative sera as control group	►The mean concentration of serum **testosterone** was statistically **higher** in *T*. *gondii*- seropositive **men and women** compared to non-infected men and women.	9
11	Borráz-León et al. [43] 2021 Mexico	●A case-control study to examine the relationship between *T*. *gondii* infection with serum testosterone, Interpersonal Sensitivity and Psychoticism symptoms among men and women	●213 healthy subjects (males = 108, females = 105) were enrolled. ●22 and 13 men and women had *T*. *gondii* IgG antibody 86 and 92 women were seronegative for *T*. *gondii* IgG antibody	►*Toxoplasma*-seropositive **men** had **higher** **testosterone** levels (*P*<0.001), Interpersonal Sensitivity (*P*<0.03) and Psychoticism symptoms (*P*<0.037) than non-infected men. ►*Toxoplasma*-infected **women** did not differ from control women.	10
12	Kadhim and AL-awadi. [34] 2013 Iraq	●A case-control study to examine the relationship between *T*. *gondii* infection with serum testosterone, progesterone and prolactin levels in pregnant women with chronic toxoplasmosis	●A total number of 55 *T*. *gondii*-IgG seropositive pregnant women and 51 seronegative pregnant women were enrolled as case and control groups, respectively	►*Toxoplasma*-seropositive **women** had a significant **higher** level of **testosterone**, but not progesterone and prolactin than *Toxoplasma*-seronegative women.	8
13	Al-Masoudi et al. [35] 2018 Iraq	●A case-control study to examine the relationship between toxoplasmosis and, testosterone and LH hormones	●66 healthy subjects were enrolled ●20 (**male** = 15 and **female** = 5) seropositive individuals were enrolled as case and the same number of healthy subjects were enrolled as control group	►A **decreased** level of **testosterone** and an **increased** level of **LH** were found in *T*. *gondii* seropositive individuals compared to control.	8
14	Al-Kurdy et al. [36] 2020 Iraq	●A case-control study to examine the relationship between toxoplasmosis and testosterone in healthy **men**	●38 *T*. *gondii* seropositive **men** and the same number of seronegative men were enrolled as the case and control groups, respectively	►**No statistical differences** were found in concentration of testosterone among the case than the control group.	8
15	AL-Asady [37] 2017 Iraq	●A case-control study to examine the relationship between toxoplasmosis with testosterone, FSH and LH in healthy pregnant women	●59 *T*. *gondii*-IgG positive **pregnant women** and 28 *T*. *gondii*-IgG negative pregnant women were enrolled as case and control groups, respectively	►The result showed **very slightly higher** serum levels of **testosterone** and **LH** and an insignificant lower level of **FSH** were detected in seropositive **women** compared to controls.	9
16	El-Gebaly et al. [38] 2019 Egypt	●A case-control study to assess seroprevalence/serointensity of toxoplasmosis in schizophrenic patients in relation to the levels of testosterone, cortisol and GSH activity	●120 schizophrenic inpatients were compared with 120 individuals attending the outpatients’ clinics	►In ***T*. *gondii*-seropositive** patients, **testosterone** was **higher** in **both genders** and **glutathione** was **lower**, while no significant difference was documented in relation to PANSS, treatment with electroconvulsive-therapy (ECT) or cortisol level. ►Schizophrenic patients showed **higher** ***Toxoplasma* antibody titer**, **cortisol**, and free **testosterone** levels in both genders and **lower** **GSH** than control.	9
117	Bayani et al. [31] 2022 Iran	●A case-control study to examine the relationship between toxoplasmosis with testosterone, prolactin, DHEA, FSH, LH, and TSH among *T*. *gondii* infected and uninfected infertile couples	●376 (188 males and 188 females) were enrolled. ●*T*. *gondii*-IgG and IgM seropositivity were detected in 56.9% (107/188) and 6.5% (7/107) of **females**, respectively. ●*T*. *gondii*-IgG and IgM seropositivity were detected in 111/188 (59.0%) and 9/111 (8.1%) of **males**, respectively.	►In **females**, **DHEA** was **lower** and the mean level of **prolactin**, **LH**, **FSH** and **TSH** were **higher** among seropositive cases compared with seronegative cases, but there were **no statistically significant differences**. ►**Testosterone** was unchanged among seropositive and seronegative **females**. ►A positive correlation was seen between toxoplasmosis and the upper and lower ranges of the normal value of **prolactin** in **females** (x 2 = 6.5, p = 0.039) but not in male cases (x 2 = 1.06, p = 0.59). ►In **males**, the mean level of **testosterone** and **TSH** were **higher** and the mean level of **prolactin** and **DHEA** were **lower** among seropositive **cases** compared with seronegative cases, but there were **no statistically significant differences**. ►A positive association was observed between *T*. *gondii* infection and the upper and lower ranges of the normal value of **testosterone** in **males** (x 2 = 6.8, p = 0.033) but not in females (x 2 = 0.62, p = 0.99).	10
18	Hagag et al. [39] 2022 Egypt	●A case-control study to examine the association of latent toxoplasmosis with testosterone levels among androgenic alopecia and acne vulgaris patients	●30 androgenic and alopecia and 30 acne vulgaris patients	►There was a statistical significance relationship between *T*. *gondii* seropositivity with androgenic alopecia severity (*P* = 0.001). ►There was a significant **elevation** of **free testosterone** in seropositive subgroups of androgenic alopecia compared with seronegative group. ►There was a statistical significance between *T*. *gondii* seropositivity with acne vulgaris severity (P = 0.019) ►There was a significant **elevation** of **free testosterone** in seropositive subgroups of acne vulgaris compared with seronegative group.	10

**2D:4D ratio:** second to fourth digit ratio, **DHEA**: dehydroepiandrosterone, **FSH:** follicle-stimulating hormone, **LH:** Luteinizing Hormone, **GSH:** Glutathione, **TSH**: thyroid stimulating hormone, **QA:** Quality Assessment.

**Table 2:** Effect of *T*. *gondii* infection on testosterone in animal models.

NO	First Author, Publication Year, and Country	Animal type	*T*. *gondii* strain	Type of inoculation	Findings	QA
1	Kanˇková et al. [49] 2011 Czech Republic	Cross-breeds of BALB/c female mice and C57 80 Black male mice of the F1 generation	Virulent strain T38 isolated from oocysts released by a stray cat	●Oral cysts ●12 female mice and 12 male mice were orally infected with brain homogenate from mice infected with cystogenic but relatively virulent strain T38 of *T*. *gondii*. ● 21 female mice and 20 male mice were given the same amount of isotonic saline (0.8% NaCl)	►Infected mice had significantly **lower** concentration of **testosterone** than controls (Tau = 0.271, *P* = 0.001). ►Both **female and male** mice with latent toxoplasmosis had sig**nificantly lower levels of testosterone** (females: Z = 2.32, P = 0.020; males: Z = 2.76, P = 0.005)	10
2	Abdoli et al. [48] 2012 Iran	Male rats (Albino Wistar type)	*RH* strain (type 1 *T*. *gondii* strain)	●Intraperitoneal injection of tachyzoites ●Case: 35 infected male rats ●Control: 21 uninfected male rats	►A temporary **decline** in serum and intratesticular **testosterone**, and **fructose** in seminal vesicle were observed. ►The rates of sperm motility (%), viability (%), and concentration were significantly decreased and sperm abnormality (%) was significantly increased after infection, but it reverts to the normal level on day 60 and 70 post infection.	9
3	Lim et al. [46] 2013 Singapore	Male Wistar rats	Prugniaud strain (type 2 *T*. *gondii* strain)	●Intraperitoneal injection of tachyzoites	►*T*. *gondii* infection **enhances testicular expression of genes involved in facilitating synthesis of testosterone** (LHR, StAR and P450scc), resulting in greater testicular testosterone production.	8
4	Afshari et al. [47] 2013 Iran	Male Wistar rats	*RH* strain (type 1 *T*. *gondii* strain)	●Intraperitoneal injection of tachyzoites ●Case: 10 infected male rats ●Control: 10 uninfected male rats	►Serum alkaline phosphatase and **testosterone** were significantly **increased** in case than control group	9
5	Abdulai-Saiku and Vyas. [51] 2017 Singapore	Female Wistar rats	Prugniaud strain (type 2 *T*. *gondii* strain)	●Intraperitoneal injection of tachyzoites	► *T*. *gondii* infection **did not change** circulating levels of **testosterone** in the blood ►*T*. *gondii* infection **did not affect** levels of serum **estrogen and progesterone** in gonadally intact females	9
6	Laubach et al. [50] 2022 USA	spotted hyenas (*Crocuta crocuta*)	-	●The relationship between *T*. *gondii* infection and plasma testosterone and cortisol levels were investigated among 109 spotted hyenas	►A negative association was found between *T*. *gondii* infection and plasma testosterone among female (cubs and subadults) and adult male hyenas, which means that the infected animals have **lower** **testosterone** levels than uninfected animals. ►No associations were found between *T*. *gondii* infection and cortisol in any age class or sex group of hyenas.	9

**LHR:** Luteinizing hormone receptor, **StAR**: Steroidogenic acute regulatory, QA: Quality Assessment.

### 3.2 Quality assessment

The results of quality assessment according to JBI for eligible studies are depicted in Tables 1 and 2. The included articles in the present meta-analysis showed an acceptable quality.

### 3.3 Description of included studies

#### 3.3.1 Human studies

In human studies, 18 articles were included (Table 1). The studies were reported from six countries, including Iran (six studies [26–31]), Iraq (six studies [32–37]), Egypt (two studies [38, 39]), and each of Czech Republic [40, 41], Romania [42], and Mexico [43] with one study (Table 1).

**Fig 2:**
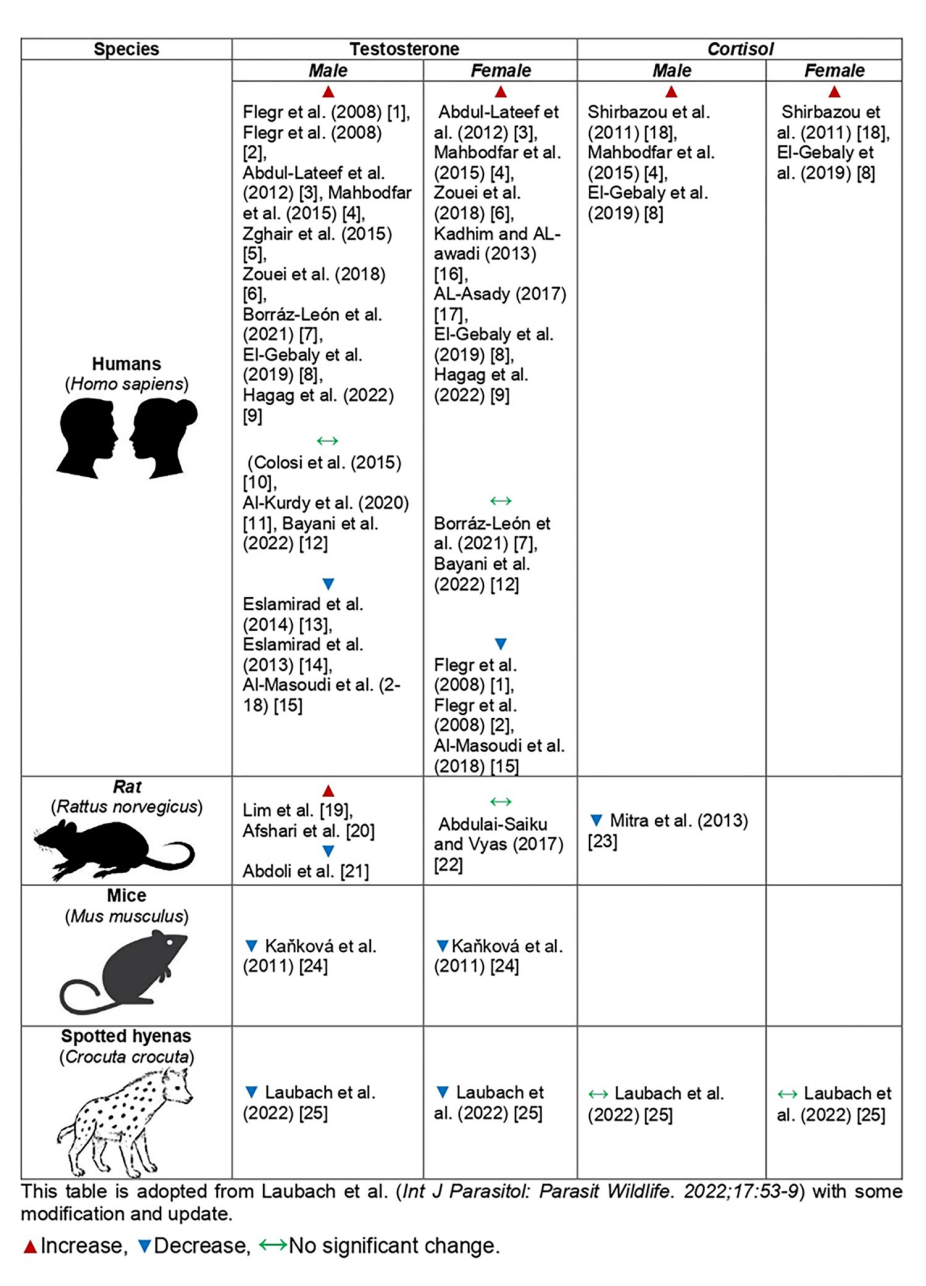
A summary of studies on the relationship between *T*. *gondii* infection, testosterone, and steroid hormone levels in males and females.

*3*.*3*.*1*.*1 Evidence for increased testosterone in human infected with T*. *gondii*. Fig 2 summarizes the included studies and Table 1 represents the details of each study. In males, 9 studies reported an increased level of testosterone in *T*. *gondii* seropositive individuals compared to seronegative counterparts [29, 30, 32, 33, 38–41, 43]. As such, in females, seven studies reported elevated levels of testosterone in *T*. *gondii* seropositive than seronegative counterparts [29, 30, 32, 34, 37–39].

*3*.*3*.*1*.*2 Evidence for a decreased or unchanged level of testosterone in human infected with T*. *gondii*. Three studies in males [27, 28, 35] and three studies in females [35, 40, 41] reported a declined level of testosterone in *T*. *gondii* seropositive than seronegative counterparts. Moreover, three studies in males [31, 36, 42] and two studies in females [31] reported no significant change in testosterone levels in *T*. *gondii* seropositive than seronegative counterparts (Table 1 and Fig 2).

*3*.*3*.*1*.*3 Evidence for increased cortisol levels in human infected with T*. *gondii*. Three studies in males [26, 29, 38] and two studies in females [26, 38] reported an increased level of cortisol in *T*. *gondii* seropositive than seronegative counterparts.

*3*.*3*.*1*.*4 Description of human studies*. The first studies regarding *T*. *gondii* and testosterone in humans were conducted by Flegr et al in 2008. In the Czech Republic [40, 41]. They conducted case-control studies among *T*. *gondii* IgG seropositive female and male students. The results showed that *T*. *gondii*- seropositive men have a higher concentration of testosterone and *T*. *gondii*- seropositive women have a lower concentration of testosterone compared with *Toxoplasma*-free subjects [40, 41]. An article was published by a group of researchers in Iran [26]. They found that *T*. *gondii* seropositive women and men had a higher concentration of serum cortisol and testosterone than seronegative individuals. As such, a significant association was found between *T*. *gondii* seropositivity with hair loss in women, hirsutism in women, and height increase in women and men. Stress and anxiety indices were also increased in *T*. *gondii* seropositive men and women, whereas the depression index increased only in seropositive men compared with the control group [26]. Abdul-Lateef et al. [32] found a significant correlation between *T*. *gondii* IgG seropositivity with an increase in serum testosterone, IL-12, and IFN-γ among an Iraqi population [32]. Eslamirad et al. [28] found an association between *T*. *gondii* IgG seropositivity with decreased testosterone levels in healthy men than the seropositivity control group [28], but they did not find an association between *T*. *gondii* seropositivity and serum lipid levels [27]. Mahbodfar et al. [29] found that *T*. *gondii* seropositive individuals had significantly higher levels of testosterone and cortisol than seronegative individuals. As such, the rates of alopecia and acne were significantly increased in seropositive men than seronegative men, and the rate of hirsutism was significantly increased in seropositive women than seronegative women [29]. Colosi et al. [42] found no statistically significant difference in serum testosterone, follicle-stimulating hormone (FSH), and sperm characteristics among *T*. *gondii* seropositive men compared with seronegative individuals. Zghair et al. [33] demonstrated that the levels of total and free testosterone, but not FSH, were significantly higher in *T*. *gondii*-seropositive men compared with the seronegative control group. Zouei et al. [30] found a statistically significant increase in the level of serum testosterone among *T*. *gondii*- seropositive men and women compared to non-infected men and women in an Iranian population. Borráz-León et al. [43] showed a significantly positive relationship between *T*. *gondii* IgG seropositivity with higher testosterone levels, interpersonal sensitivity, and psychoticism symptoms in seropositive men, but not women, than non-infected control groups [34]. A study among *T*. *gondii* seropositive and seronegative women revealed an increased level of testosterone, but not progesterone and prolactin, in seropositive women compared with seronegative control groups. Al-Masoudi et al. [35] found a decreased level of testosterone and an increased level of luteinizing hormone (LH) in *T*. *gondii* seropositive individuals compared to controls in a healthy Iraqi population. Al-Kurdy et al. [36] found no statistical differences in the concentration of testosterone among the *T*. *gondii* seropositive men than seronegative controls. AL-Asady et. Al. [37] found a very slightly higher serum level of testosterone and LH and insignificant lower levels of FSH in seropositive women compared to controls. El-Gebaly et al. [38] demonstrated that schizophrenic patients showed higher *T*. *gondii* antibody titer, cortisol, and free testosterone levels in both genders and lower Glutathione (GSH) than controls. As such, *T*. *gondii* seropositive schizophrenic patients had higher testosterone levels and lower glutathione levels than seronegative patients. Bayani et al. [31] investigated the relationship between toxoplasmosis with testosterone, prolactin, dehydroepiandrosterone (DHEA), FSH, LH, and thyroid stimulating hormone (TSH) among *T*. *gondii* seropositive and seronegative infertile couples. Although some alterations were observed, no statistically significant differences were detected in these hormones among *T*. *gondii* seropositive and seronegative groups [31]. In an interesting report, Hagag et al. [39] found a positive association between *T*. *gondii* seropositivity and a significant elevation of free testosterone levels among patients with androgenic alopecia and acne vulgaris compared with the seronegative group. There are also some case reports regarding the association of acute toxoplasmosis with lower testosterone levels in males with hypogonadotrophic hypogonadism [44] as well as a case with intracranial toxoplasmosis presenting as panhypopituitarism [45].

**Fig 3:**
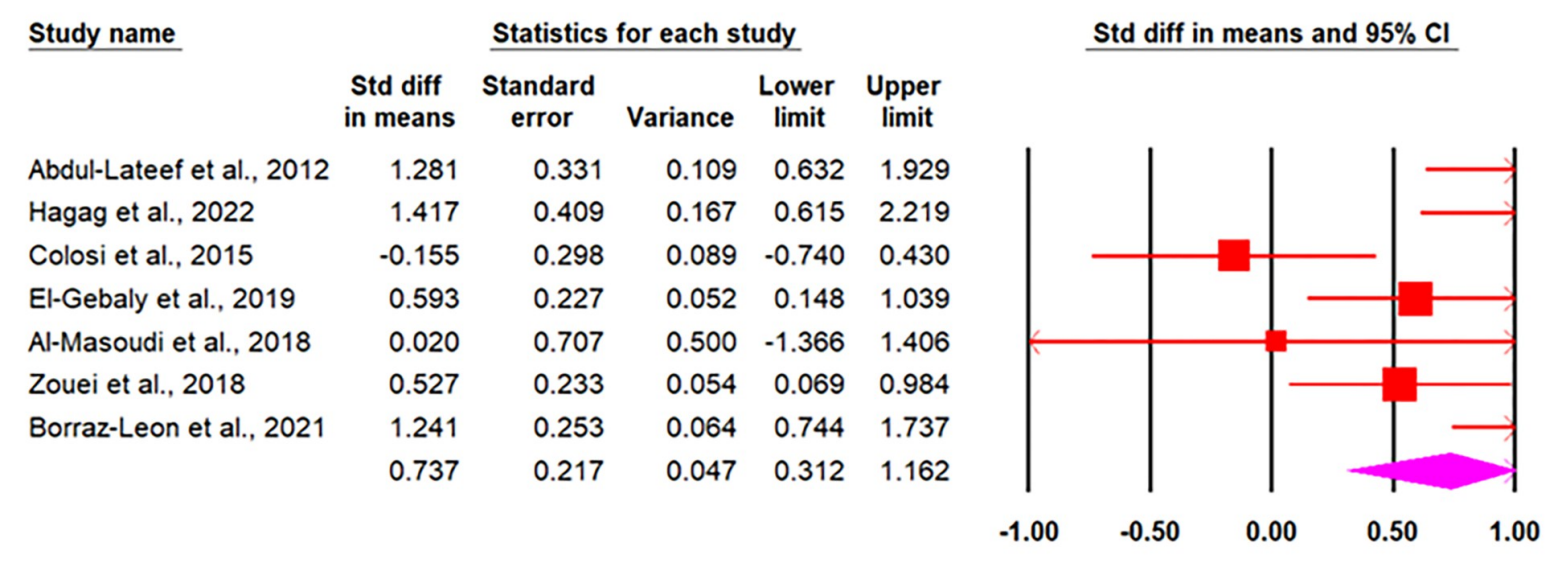
Forest plot of the pooled mean± SD of testosterone in *T*. *gondii* positive than *T*. *gondii* negative in males, estimated with random-effects model.

**Fig 4:**
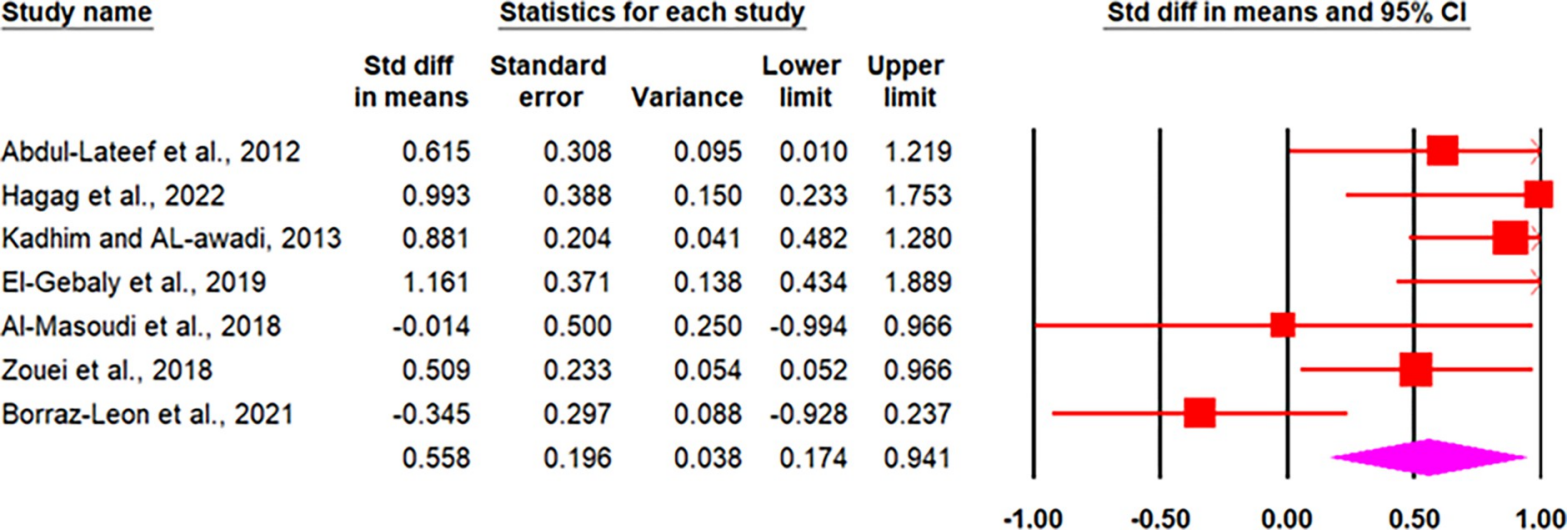
Forest plot of the pooled mean± SD of testosterone in *T*. *gondii* positive than *T*. *gondii* negative in females, estimated with random-effects model.

*3*.*3*.*1*.*5 Meta-analysis of human studies*. As shown in Table 3, eleven papers (seven datasets in males and seven datasets in females) on the association between *T*. *gondii* and testosterone were eligible to include in the data synthesis. Based on the random-effects model, the pooled mean± SD of testosterone in *T*. *gondii* positive than *T*. *gondii* negative were calculated to be 0.73 and 0.55 in males and females, respectively (Figs 3 and 4). It means that, testosterone increased by 0.73 and 0.55 units in *T*. *gondii* positive compared to *T*. *gondii* negative males and females, respectively. The publication bias was not statistically significant in males (*p* = 0.95) and females (*p =* 0.71), respectively.

**Table 3:** Included studies on the association between *T*. *gondii* positive and *T*. *gondii* negative with testosterone.

First author	Gender	*Toxoplasma* positive			*Toxoplasma* negative			*P*-value
		Total sample size	Mean (ng/ml)	St.Deviation	Total sample size	Mean (ng/ml)	St.Deviation	
**Abdul-Lateef et al., 2012 [32]**	**Male**	37	8.0601	3.04751	15	4.1123	3.17078	0
**Abdul-Lateef et al., 2012 [32]**	**Female**	40	0.7213	0.35507	15	0.5249	0.18708	0.011
**Bayani et al., 2022 [31]**	**Mixed †**	99	0.6	0.5	71	0.6	0.5	0.9
**Hagag et al., 2022 [39]**	**Male**	14	28.01	12.95	16	13.62	6.86	0.001
**Hagag et al., 2022 [39]**	**Female**	14	11.98	14.26	16	2.3	1.04	0.001
**Kadhim and AL-awadi., 2013 [34]**	**Female**	55	1.95	1.37	51	0.94	0.84	1.80E-05
**Colosi et al., 2015 [42]**	**Male**	15	399.07	185.18	45	425.96	170.05	0.62
**El-Gebaly et al., 2019 [38]**	**Male**	42	10.8	6.23	39	7	6.59	0.01
**El-Gebaly et al., 2019 [38]**	**Female**	12	8.5	9.62	27	2.2	1.65	0.003
**Al-Masoudi et al., 2018 [35]**	**Male**	4	0.85	6.25	4	0.73	5.95	NR
**Al-Masoudi et al., 2018 [35]**	**Female**	8	0.3	0.87	8	0.31	0.54	NR
**Mahbodfar et al., 2015 [29]**	**Mixed †**	119	5.83	5.39	96	3.38	3.92	0
**Zouei et al., 2018 [30]**	**Male**	38	5.6	1.99	38	4.56	1.96	NR
**Zouei et al., 2018 [30]**	**Female**	38	0.41	0.22	38	0.31	0.17	NR
**Zghair et al., 2015 [33]**	**Mixed †**	121	6.515	0.51	30	6.78	0.61	NR
**Borráz-León et al., 2021 [43]**	**Male**	22	7.78	2.66	86	4.32	2.82	<0.001
**Borráz-León et al., 2021 [43]**	**Female**	13	0.63	0.37	92	1.18	1.69	0.49

**†** Not included in Meta-analysis.

#### 3.3.2 Animal studies

*3*.*3*.*2*.*1 Evidence for increased levels of testosterone in animals infected with T*. *gondii*. Two studies in rats [46, 47] reported an increased level of testosterone in infected than non-infected animals (Fig 2 and Table 2).

*3*.*3*.*2*.*2 Evidence for decreased or unchanged levels of testosterone in animals infected with T*. *gondii*. Three studies in male animals (rats [48], mice [49], and spotted hyenas [50]) and two studies in female animals (mice [49], and spotted hyenas [50]) reported a decline level of testosterone in infected animals compared with non-infected animals. While, one study in female rats [51] reported no significant changes of testosterone in infected than non-infected animals (Fig 2 and Table 2).

*3*.*3*.*2*.*3 Evidence for alteration of cortisol levels in animals infected with T*. *gondii*. One study reported a declined level of cortisol in *T*. *gondii-*infected male rats [52], while one study [50] reported no significant change of cortisol levels in *T*. *gondii-*infected male and female spotted hyenas.

*3*.*3*.*2*.*4 Description of animal studies*. Kanˇková et al. [49], reported that *T*. *gondii*-infected mice (both females and males) had significantly lower concentration of testosterone. Abdoli et al. [48] reported that male rats with *T*. *gondii* infection had a temporary decline in serum and intratesticular testosterone and fructose in seminal vesicles. As such, the percentage rates of sperm motility, viability, and concentration were significantly decreased and sperm abnormality was significantly increased after infection, but it reverts to the normal level on days 60 and 70 post infection [48]. Lim et al. [46] observed that *T*. *gondii* infection in male rats enhances testicular expression of genes involved in the synthesis of testosterone (LHR, StAR, and P450scc), resulting in greater testicular testosterone production. Afshari et al. [47] showed significantly increased levels of serum alkaline phosphatase and testosterone in *T*. *gondii*-infected male rats compared with the uninfected control group. Laubach et al. [50] found a negative association between *T*. *gondii* infection and plasma testosterone among female (cubs and subadults) and adult male hyenas, which means that the infected animals have lower testosterone levels than uninfected animals. Indeed, no associations were found between *T*. *gondii* infection and cortisol in any age class or sex group of hyenas [50].

## 4. Discussion

Testosterone is involved in a variety of physiological functions, such as behavioral traits and reproductive functions in both sexes [21]. In this study, we reviewed data regarding *T*. *gondii* infection and testosterone variations in human studies and animal models (Fig 2 and Tables 1 and 2). We observed that most of the included studies in humans reported an increased level of testosterone [26, 29–34, 37–41, 43], while some studies reported a decreased level [27, 28, 35] or insignificant changes [36, 42] (Fig 1). As such, these variations were different in males and females in some studies [40, 41]. In animal models, some studies reported a declining level of testosterone [48–50], while others reported an increased level [46, 47] or insignificant changes [51]. Notably, variations in testosterone levels are most probably due to infection with different parasite strains, or a difference in host variations, which consequently influence the intensity of infection [15, 16, 22, 53]. Host variations also influence the intensity of *T*. *gondii* infection [54]. Among animals, mice and New and Old-World monkeys are highly sensitive to *T*. *gondii* infection; while sheep are intermediately sensitive, and goats, cattle, deer, horses, and pigs are resistant to the infection [54]. In humans, immunocompromised patients and pregnant women are at high risk of severe *T*. *gondii* infection, while *T*. *gondii* infection is usually asymptomatic (latent) among immunocompetent individuals, [7, 12]. Like humans, the laboratory rat (*Rattus norvegicus*) is resistant to *T*. *gondii* infection and is a suitable model for the study of chronic *T*. *gondii* infection [55, 56].

Testosterone plays an important role in sexual behavior and mating success [57–60]. On the other hand, recent evidence revealed that *T*. *gondii* infection augments sexual behavior and attractiveness in humans [61] and experimentally infected rodents [5]. In this regard, Borráz-León et al. [61] assessed several factors related to attractiveness among *T*. *gondii*-infected and non -infected individuals. They found that both *T*. *gondii*-infected men and women had lower facial fluctuating asymmetry, while infected women had lower body mass index, higher number of sexual partners, and a higher self-perceived attractiveness than non-infected control groups. They also assessed the attractiveness and perceived health of facial pictures of *T*. *gondii*-infected and non-infected subjects by an independent group of raters and found that both infected women and men were rated as more attractive and healthier than non-infected individuals [61]. Increased testosterone could enhance sexual behavior and attractiveness in infected subjects and could increase mating opportunity and transmission of *T*. *gondii* through sexual intercourse. In this regard, Lim et al. [46] reported that *T*. *gondii* infection (induced by Prugniaud strain) enhances testicular expression of genes that are involved in the synthesis of testosterone in experimentally infected male rats. Dass et al. [5] demonstrated that *T*. *gondii*-infected male rats had higher sexual attractiveness to non-infected females, resulting in increased mating of infected males with non-infected females. They also confirmed sexual transmission of *T*. *gondii* through intercourse, whereas *T*. *gondii* cysts were detected in the epididymis of infected males, vaginal lavage of naïve females that mated with infected males, as well as in brains of pups which born from these matings [5]. As such, secretion of *T*. *gondii* in semen and sexual transmission of the parasite have been reported in dogs [62], goats [63–65], sheep [66, 67], cattle [68], and pigs [69]. Notably, *T*. *gondii* transmission in sheep was reported by artificial insemination of contaminated frozen semen [70]. There is also indirect evidence that suggests sexual transmission of *T*. *gondii* in humans. In this regard, a recent study by Tong et al. [4] confirmed the presence of *T*. *gondii* tissue cysts in human semen by immunofluorescence staining and molecular methods. Furthermore, it is proposed that unprotected sex and oral sex could be an important route of *T*. *gondii* transmission in humans [71, 72]. Hlaváčová et al. [73] performed a two-year study to compare the seropositivity to *T*. *gondii* in couples and analyzed the serological status of sexual partners. The results indicated that the prevalence of *T*. *gondii* infection was higher in women who had infected male partners than in women with uninfected male partners (25.6% *vs* 18.2%, respectively; *P* = 0.045). This study also suggests that a partner’s seropositivity may be a risk factor for infection in women (prevalence ratio = 1.418; *P* = 0.045) but not in men (prevalence ratio = 1.058; *P* = 0.816) [73]. This evidence was also supported by studies among female sex workers [74] and individuals with a history of sexual promiscuity [75] in Mexico. In this regard, Alvarado-Esquivel et al. [74] found a significantly higher incidence of latent toxoplasmosis among female sex workers compared with age- and sex-matched control groups (15.44% *vs* 3.67% in case and control groups, respectively, *P* = 0.0001). As such, female sex workers had significantly higher anti-*T*. *gondii* IgG titers (>150 IU/mL) than the control group (9.6% *vs* 2.9%, respectively *P* = 0.007) [74]. Another study by the same group of researchers in Mexico [75] revealed a significantly higher prevalence of anti-*T*. *gondii* IgG antibodies among individuals with sexual promiscuity than individuals without this practice (18.1% *vs* 10.3%, respectively; OR: 1.91; 95% CI: 1.41–2.60; *P*< 0.0001). Indeed, higher titers of anti-*T*. *gondii* IgG antibodies (>150 IU/mL) were significantly increased in participants with sexual promiscuity than participants without this history (9.2% *vs* 4.6% respectively; OR: 2.09; 95% CI: 1.38–3.16; *P* = 0.0003). Additionally, the association of *T*. *gondii* seropositivity and serointensity with sexual promiscuity was observed in men but not in women [75]. Collectively, it seems that *T*. *gondii* infection could manipulate the mate choice of their host to increase their transmission rates. This phenomenon could be mediated partly by enhancing testosterone levels, which consequently increase sexual behavior and mating success [22, 76].

Testosterone has a pivotal role in spermatogenesis and male reproductive functions. A declined level of testosterone was reported following *T*. *gondii* infection in mice [49] and rats following infection with a *T*. *gondii* type I strain [48], as well as male and female spotted hyenas (*Crocuta crocuta*) which were naturally infected with *T*. *gondii* [50]. On the other hand, *T*. *gondii* infection could induce male reproduction impairment by interfering in spermatogenesis and testicular damage [44, 48, 77–80], which may be partly mediated by declining testosterone levels. In this regard, Abdoli et al. [48] showed that *T*. *gondii* infection (induced by RH strain) induced a temporary decline in serum and intratesticular testosterone levels, fructose in seminal vesicles, as well as declining of sperm motility, viability, concentration, and increased of sperm abnormality in male rats. Hlaváčová et al. [81] compared the prevalence of latent toxoplasmosis in men with and without semen abnormalities and found that *T*. *gondii*-infected men had significantly lower sperm concentration and motility compared with *T*. *gondii*-negative men. Although another human study did not find a significant association between latent toxoplasmosis and semen abnormalities [42]. Considering the possible role of *T*. *gondii* in male reproductive impairment, it is recommended that populations with high prevalence of male infertility be examined for *T*. *gondii* infection.

Testosterone has also a pivotal role on behavioral traits in males and females, such as aggressive behavior [22, 82–84]. On the other hand, latent toxoplasmosis is also involved in the etiopathogenesis of different behavioral alterations (e.g., psychoticism [43], aggressive behavior [85, 86], and violent behavior [87]) and neuropsychiatric diseases, such as schizophrenia [88, 89], depression [90, 91] and anxiety disorders [90, 92, 93], obsessive compulsive disorder (OCD) [94], and autism spectrum disorder (ASD) [95–98]. Different mechanisms have been proposed to be involved in the etiopathogenesis of these disorders following *T*. *gondii* infection, including CNS Inflammation [99, 100], neurotransmitter alterations (alterations in dopamine [101–106] and serotonin synthesis [91]) and testosterone alteration [22, 107]. On the other hand, *in vitro* experiments revealed that testosterone [108] and dopamine [109] stimulate the propagation of *T*. *gondii* tachyzoites *in vitro*. Increasing fetal testosterone is also involved in autistic traits [110–113]. It is an important point because toxoplasmosis is a worldwide prevalent infection [114]. It is plausible an increased risk of ASD among infants of mothers with latent toxoplasmosis, and this phenomenon may partly be mediated via maternal testosterone alteration in mothers with latent toxoplasmosis [95].

There are some limitations to this systematic review. The lack of published articles from many countries were infertility is common is a major limitation. The observed association should be interpreted with caution, because the timeline of *T*. *gondii* infection and disease process could not be evaluated from the available data. Importantly, *T*. *gondii* seroprevalence has been associated with many different risk factors, which were not evaluated in this work. As such, such confounding factors, including environmental toxins [115–117] and coinfections with other pathogens [100] may also affect the levels of sex hormones.

The results of this work can provide useful guidance for planning future studies. It would be important to focus on those parts of the world in which there is a lack of data on this subject. Moreover, including all pertinent risk factors would allow to better clarify the epidemiological aspects of *T*. *gondii* infection in infertility individuals and testosterone alterations. Optimally, prospective cohort studies and using more comprehensive serology panels (e.g., including IgG avidity testing) for estimating the timing of *T*. *gondii* infection could elucidate the timeline of risk factors of infection.

## 5. Conclusion

This study indicated that latent toxoplasmosis is associated with increased testosterone levels in most studies in humans and some studies in non-human animals. This change could be associated with increased sexual attractiveness in infected subjects which lead to sexual transmission of the parasite. On the other hand, some studies demonstrated a decreased level of testosterone in *T*. *gondii*-infected animals and humans. This change could partly be associated with male reproductive impairments, which were observed in *T*. *gondii*-infected human and non-human animals. These findings suggest the great need for more epidemiological and experimental studies in depth understanding the relationship between *T*. *gondii* infection, testosterone alteration, and further consequences.

## Supporting information

S1 Checklist PRISMA 2020 checklist. (DOCX)

## Abbreviations

ASD: autism spectrum disorder
CNS: central nervous system
DHEA: dehydroepiandrosterone
FSH: follicle-stimulating hormone
GSH: Glutathione
T. gondii: *Toxoplasma gondii*
LH: Luteinizing Hormone
MeSH: Medical Subject Heading
OCD: obsessive compulsive disorder
PRISMA: The Preferred Reporting Items for Systematic reviews and Meta-Analyses
2D:4D ratio: second to fourth digit ratio
